# Supplementary figures and images for: Plant virus movement proteins originated from jelly-roll capsid proteins
Source: PLoS Biol. 2023 Jun 15;21(6):e3002157. doi: 10.1371/journal.pbio.3002157 (PMC10306228; doi:10.1371/journal.pbio.3002157)

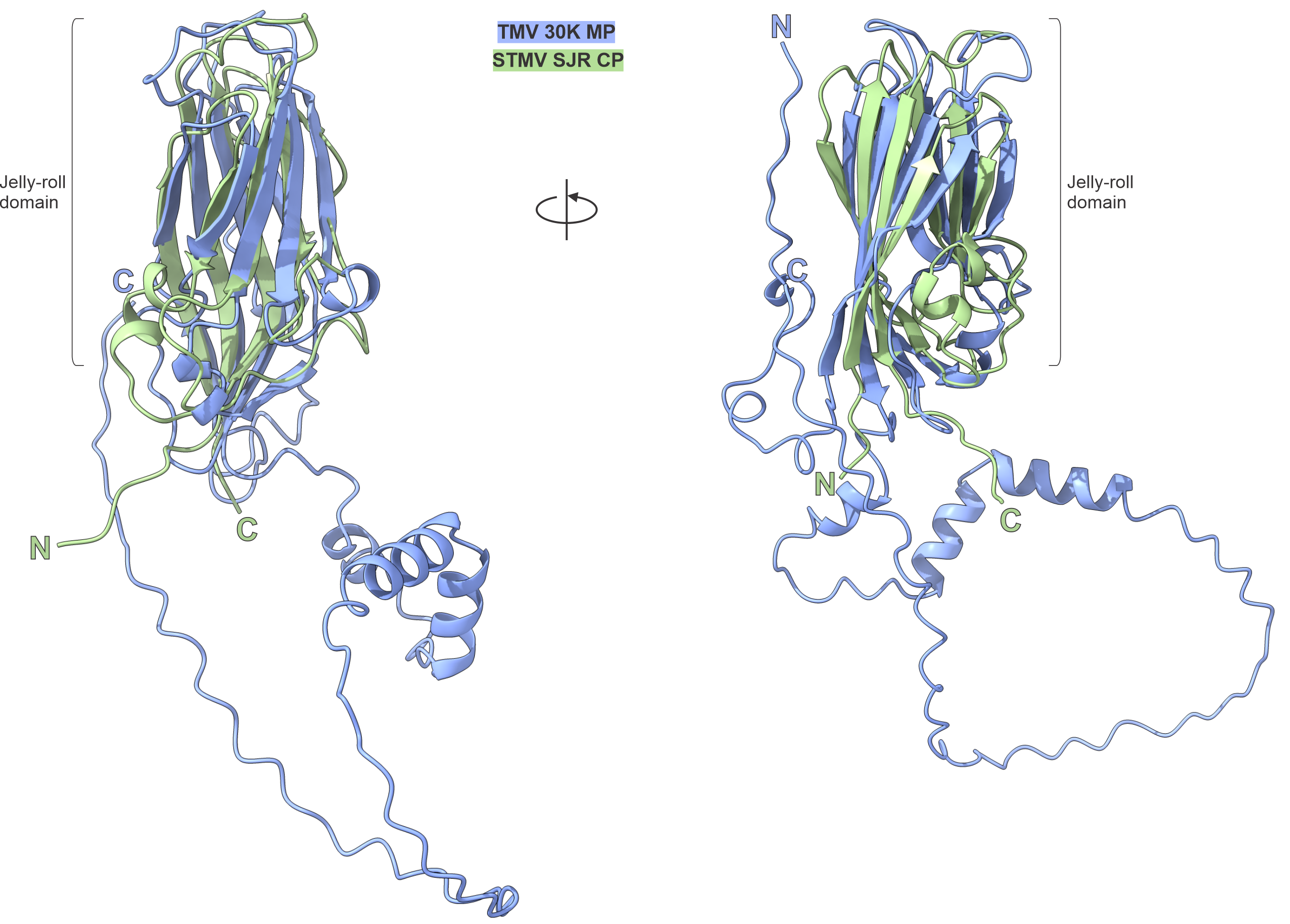

Supplement: S2 Fig — (TIF) [file pbio.3002157.s011.tif]

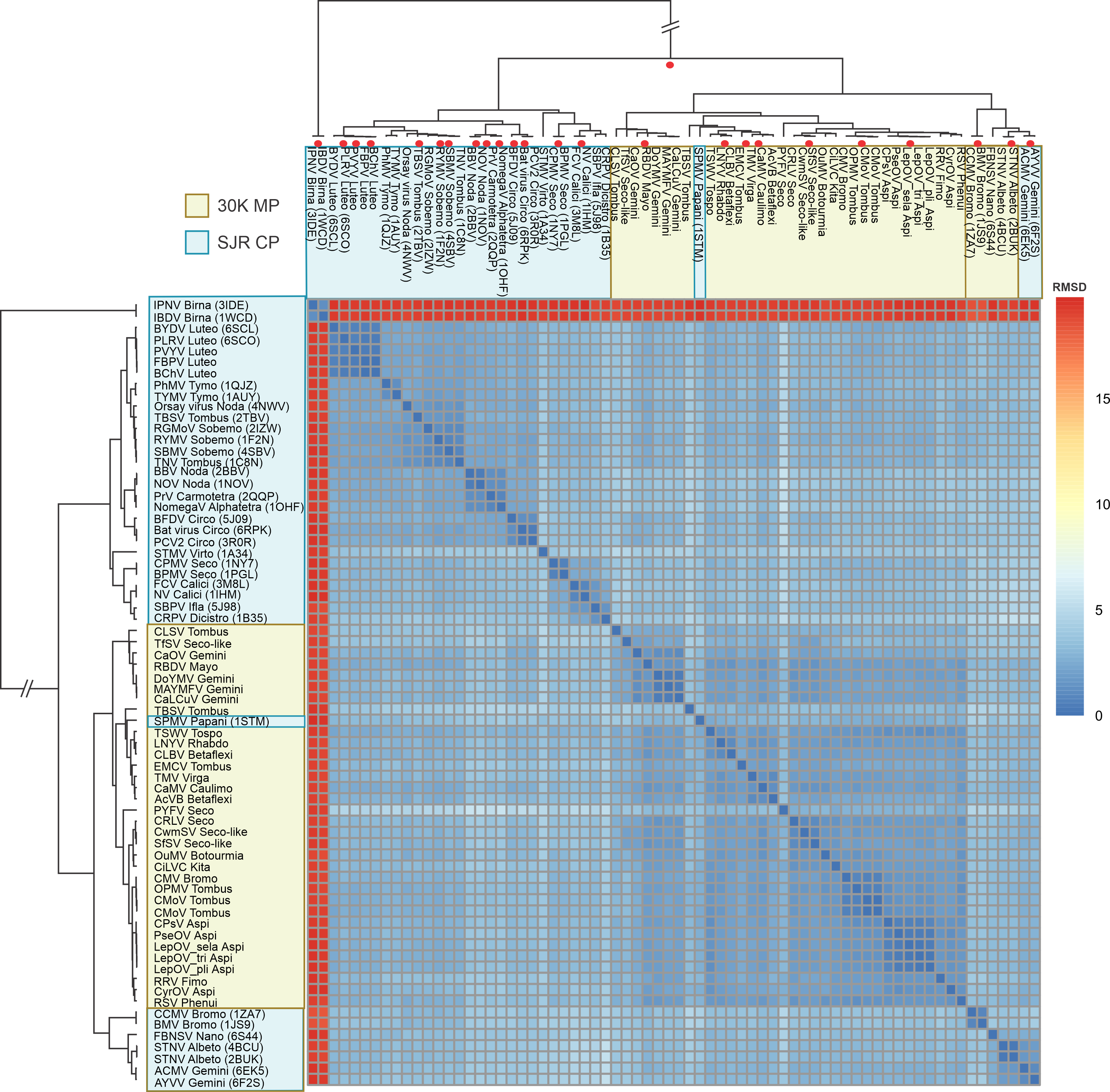

Supplement: S3 Fig — The red circles indicated in the top dendrogram, represent bootstrap values ≥90 obtained with R package “pvclust.” The CPs and MPs are indicated in blue and yellow, respectively. (TIF) [file pbio.3002157.s012.tif]

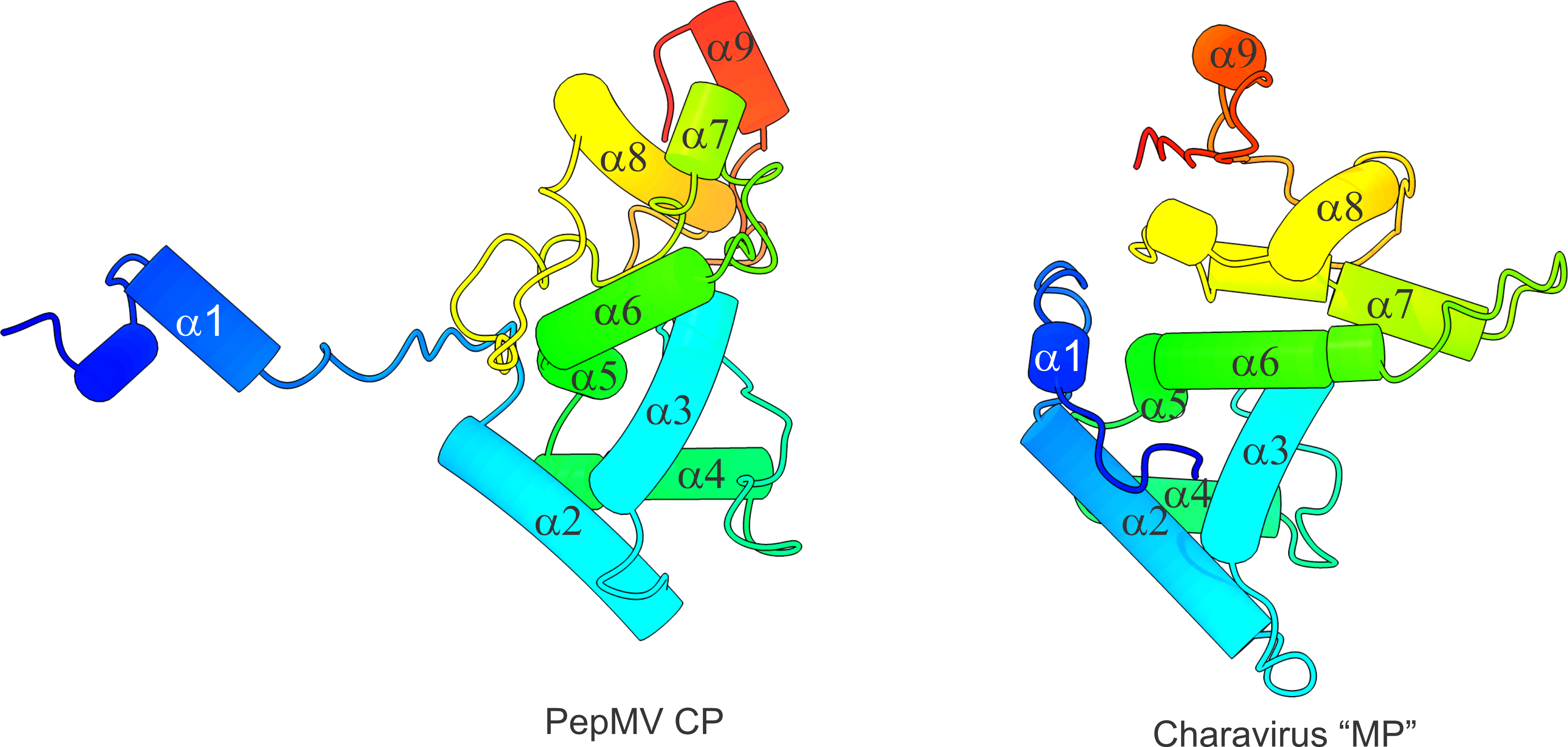

Supplement: S4 Fig — The structures are colored using the rainbow scheme from blue (N-terminus) to red (C-terminus) and α-helices equivalent between the 2 proteins are numbered. For the charavirus protein, only the region corresponding to the PepMV CP is shown. (TIF) [file pbio.3002157.s013.tif]

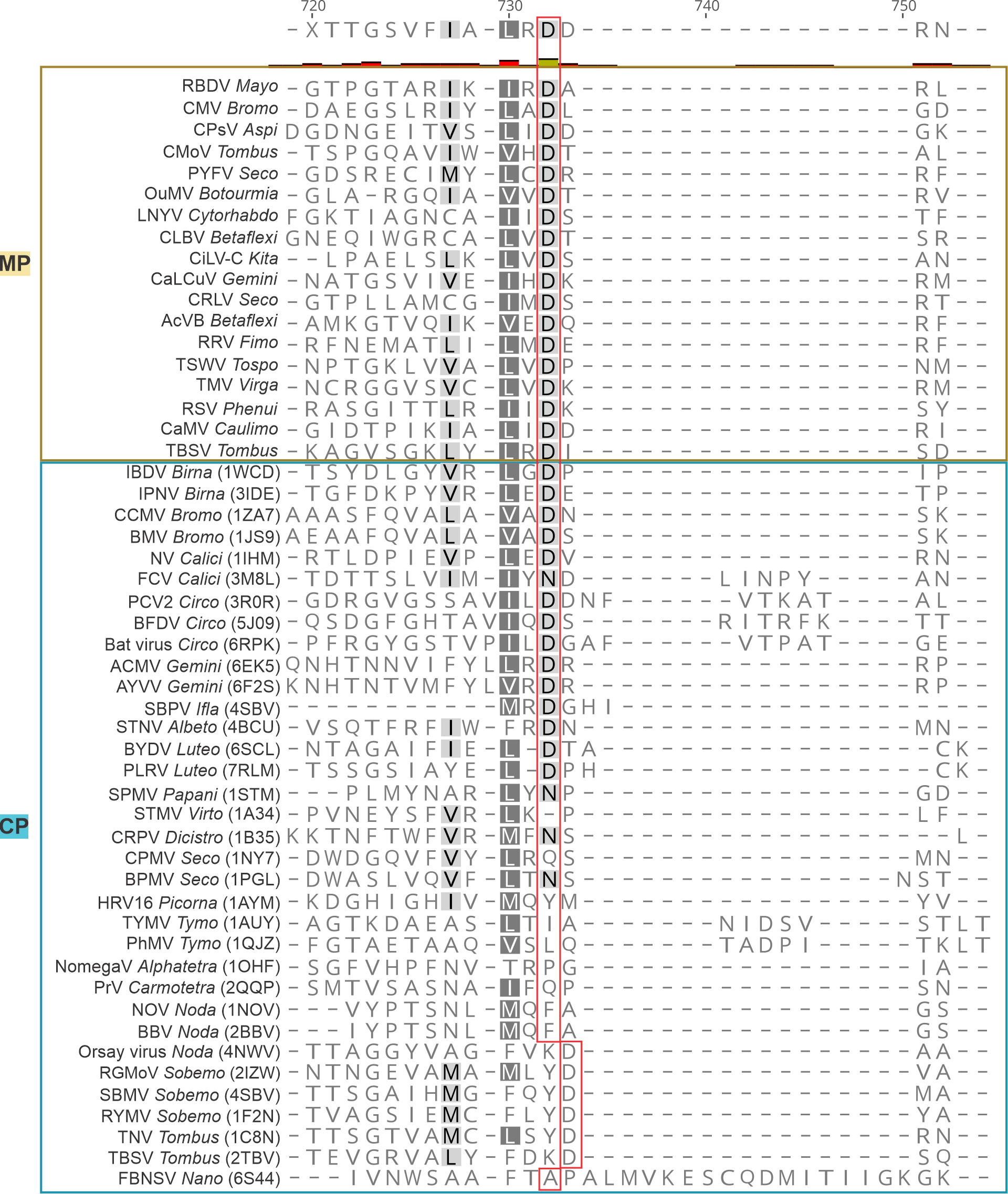

Supplement: S5 Fig — The alignment was made using PROMALS3D. Only the region encompassing the D-motif is shown. (TIF) [file pbio.3002157.s014.tif]

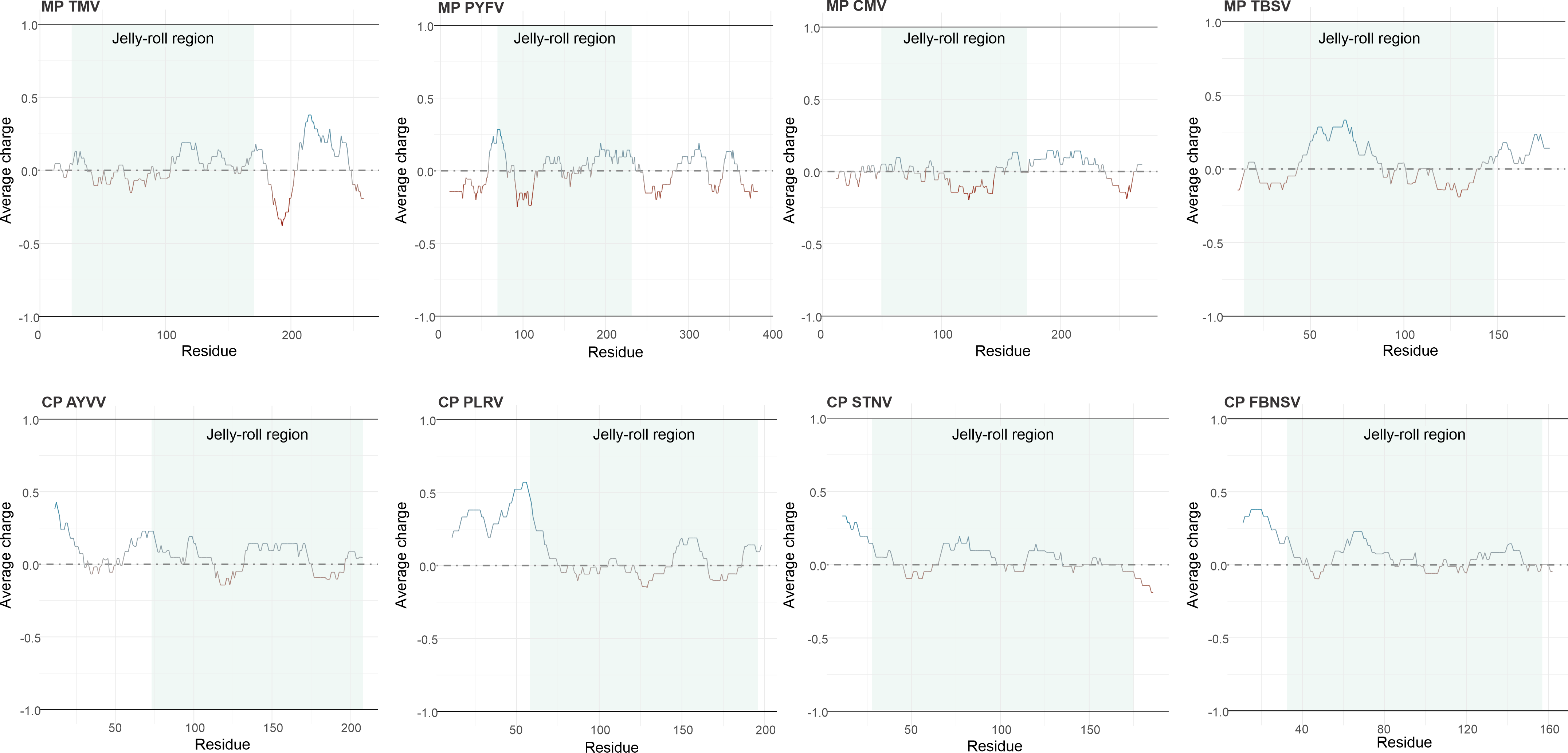

Supplement: S6 Fig — The jelly-roll region is marked in light green. (TIF) [file pbio.3002157.s015.tif]
