# Supplementary material for: Plant virus movement proteins originated from jelly-roll capsid proteins
Source: PLoS Biol. 2023 Jun 15;21(6):e3002157. doi: 10.1371/journal.pbio.3002157 (PMC10306228; doi:10.1371/journal.pbio.3002157)
Supplement: Attachment — Submitted filename: 30K_MP_rebuttal.pdf [file pbio.3002157.s016.pdf]

Editorial comments:
As you will see below, the reviewers find your work interesting but they raise several issues that should be solved before further consideration. In particular, we think it is very important that you show the confidence metrics for both AlphaFold and Rosetta, tone down statements as needed and reorganize the manuscript as needed. Both reviewers agree that you should be careful with your conclusion that geminivirus CP is an origin of MPs. Further, reviewer #2 considers that to draw a conclusion, some careful considerations on the predicted structures and the alignment are required, and this reviewer provides suggestions for data analysis to achieve this.

RESPONSE: We highly appreciate the constructive comments by both reviewers and have revised our manuscript taking all of them into consideration. In particular, we now provide the per-residue local distance difference test (lDDT) plots for both AlphaFold and RoseTTAFold models in new Supplementary Figure 1. In addition, we provide all models generated in our study in Supplementary Data 1. The statements on the origin of MPs in geminiviruses were toned down both in the text and Figure 6. We have also combined Results and Discussion, as suggested by Reviewer 2. Finally, we performed alignments of capsid and movement protein structures and calculated the corresponding tree using MUSTANG, as suggested by Reviewer 2. These new results fully support our original conclusions. We also note that while the manuscript was under review, genomes of several viruses associated with lower vascular plants (ferns) and non-vascular plants (mosses and liverworts) were reported (Viruses 2023, 15, 840; https://doi.org/10.3390/v15040840). Analysis of their 30K MPs were also included in the revised manuscript. Our point-by-point answers to the reviewers' comments are appended below.

RPOINT-BY-POINT RESPONSES TO THE REVIEWERS' COMMENTS

Reviewer #1:

The 30K family is the most prevalent among the movement proteins (MPs) of extremely diverse plant viruses that affect a broad variety of wild and crop plants alike. Despite the decades-long effort, however, the molecular mechanisms whereby 30K MPs empower plant virus infection remain poorly understood. In this respect, the work by Butkovic et al. represents a sea change by revealing 3D structures of a broad variety of 30K MPs. Methodologically, the authors used the most advanced bioinformatics available for the prediction and comparison of protein structures (AlphaFold2, RoseTTAFold, DALI), as well as a variety of more traditional, complementary approaches for the sequence and phylogenetic analyses. Furthermore, they generated a comprehensive database of 30K MPs that provides an important resource for the entire plant virology community.

This work compellingly demonstrates the single jelly-roll (SJR) fold of the structural core of all 30K MP subfamilies. The authors also analyzed additional structural elements, such as N- and C-terminal core extensions in the 30K MPs of distinct subfamilies. Taken together, the findings of this study are certain to dramatically facilitate experimental analysis of the structure-to-function relationships within 30K MPs.

Finally, this work reports a largely unexpected evolutionary discovery: the origin of the 30K MPs from the SJR capsid proteins of the icosahedral RNA and DNA viruses, representing an example of the functional repurposing of the virus proteins during expansion to a novel ecological niche.

RESPONSE: We thank the reviewer for the kind words and useful suggestions.

What this reviewer is a bit less enthusiastic about, is the suggestion that the geminiviruses could be an original virus lineage in which SJR CP gene duplication and re-functionalization have occurred. From the studies of the global distribution of virus families, it appears that the host range and geography of geminiviruses were historically limited to mostly tropical areas. This distribution has dramatically expanded into temperate regions in the second part of the XX century, likely due to the expansion of the geographical range of the whiteflies (most common geminivirus vectors) along with global warming. Accordingly, it seems unlikely that the ssDNA geminiviruses which represent a relatively minor part of the plant virome were the original source for 30K MP emergence. Because the significant majority of plant virus families possessing diverse lineages of 30K MPs are RNA viruses, it seems more plausible that these MPs have emerged among the RNA viruses.

RESPONSE: We agree with the reviewer that the evidence of the origin of 30K MPs in geminiviruses is not very strong. In the revised version of the manuscript, this claim has been toned down (in the text and figure depicting the evolutionary scenario) and the alternative possibility, namely, the more parsimonious emergence of the MP in RNA viruses has been presented.

Reviewer #2:

Butkovic et al has studied structural similarity of a jelly-roll motif and evolutionary lineage between movement proteins (MPs) encoded in broadly RNA/DNA viruses (especially in plant viruses) and capsid proteins (CPs) in ssRNA and ssDNA viruses. The results largely depend on recently invented AI-based accurate structural predictions of structurally unrevealed MPs using AlphaFold2 and RoseTTAFold. Based on the authors' structure-based alignments' and similarity analysis, previously recognized conserved secondary structure and D-motif in MPs and CPs are deeply and comprehensively analyzed, which could explain their same evolutionary origin. Butkovic et al also hypothesizes likely evolutionary scenario of a MP gene acquisition from a CP gene.

The addressed question is very interesting in the sense of understanding function and origin of the 30K MPs superfamily in diverse viruses and the hypothesized evolutionary scenario is likely. Since the jelly-roll fold and D-motif of the MPs has already mentioned in previously papers, the main contribution of the manuscript has been the thoroughly analysis of the predicted MPs and CPs structures in diverse viruses. However, the main bottleneck is no experimental structure of the MP available for confirming the hypothesis and the results. Although I agree that Al-based structural prediction is a strong and useful tool for performing structure-based classifications, it should be cautiously of validating and interpreting the results owing not to being misled by the predicted models. Therefore, major and minor comments to be addressed.

RESPONSE: We thank the reviewer for the constructive comments and suggestions.

Major and technical issues

1. Many parts of discussion and speculations are found in Results section and lengthy, and thus it is hard to read. I highly recommend reorganizing "Results" and "Discussion" or using an option of "Results and Discussion". The same or similar discussions are also found both in results and in discussion sections. It should be integrated to one.

RESPONSE: We have combined the Results and Discussion, as suggested, and hope that the revised text is now more balanced.

2. Fig. 2A and lines 196-205. The most difficult part for me to review is that there is no available data for clarifying the accuracy of the MPs' structural prediction. AlphaFold2 automatically generates per-residue pLDDT score (e.g. Tunyasuvunakool et al., 2021, DOI: 10.1038/s41586-021-03828-1) to validate predicted and generated structures. The jelly-roll core of the MPs seems to be well predicted, however most likely loops, N-/C-terminal parts are not. Such validation data should be included in the manuscript and described in the main text.

RESPONSE: Thank you for raising this valid point. We have now provided plDDT plots for all analyzed models as a new supplementary figure 1. As we originally pointed out in the text, the N- and C-terminal regions are largely unstructured and the quality of the models in these regions is poor. Hence, the structural details of these regions were considered in neither the original nor revised version of the manuscript. The model quality assessment is described in the main text, as requested by the reviewer.

Also, it is mentioned that RoseTTAFold was used in case AlphaFold2 showed poor IDDT results, but then how to verify the RoseTTAFold models were accurate?

RESPONSE: The end-to-end version of RoseTTAFold provides structural models with estimated residue-wise CA-lDDT in the B-factor column. The lDDT plots for RoseTTAFold and AlphaFold models are now provided in the supplementary information (new Supplementary Figure 1). In addition, we provide all models generated in our study in Supplementary Data 1.

3. Fig. 3 and lines 206-238. It is not clear which part of the predicted structures are used for DALI calculation. If you used the entire predicted structures, the obtained DALI-score largely affected by badly predicted loops or N- and C-terminal regions. This is crucial for discussing the structural similarity in the MPs and the CPs.

RESPONSE: We apologize for not making this clearer in the original version of the manuscript. For the dendrogram calculation with DALI, only the jelly-roll domain was considered, with the N-terminal regions which do not have counterparts in the capsid proteins being removed prior to comparison. Thus, all reported Z scores correspond to the pair-wise comparisons of the jelly-roll domains.

Could you generate a structure-based phylogeny using only accurately predicted jelly-roll domain of the MPs and the CPs? Also, DALI is a good start for identifying similar structures, but not suitable for generating accurate multiple structural alignments. There is a better alignment tool (e.g. HSF, SHP, MUSTANG...) to generate an RMSD-based phylogeny as seen in previously published papers (e.g. Riffel et al., 2002, DOI: 10.1016/S0969-2126(02)00896-1; Wang et al., 2014, DOI: 10.1038/nature13806). It should be very careful of concluding that geminivirus CP is an origin of MPs from the predicted structures.

RESPONSE: Thank you for this suggestion. In the revised manuscript, we used MUSTANG to generate the RMSD-based tree of SJR CPs and MPs. The tree is largely congruent with the dendrogram based on the DALI Z-scores, with minor differences. Essentially, as previously, MPs emerge from within the CP diversity and form a sister group to CPs of plant RNA and DNA viruses (geminiviruses, nanoviruses, bromoviruses, satellite viruses). The only difference is that the CP of SPMV is nested among the MPs. This is likely due to poor representation of this CP group (only one structure) and its simple organization which indeed closely resembles that of MPs. We discuss these findings in the revised text, and the MUSTANG tree is now provided as a new supplementary figure 3.

4. Fig. 5B and lines 301-305. It is very risky to say a function of the D-motif in the predicted structure. For me, it is not clear why this amino acid residue on loop or turn between two beta-sheets is important for protein folding. Then, why amino acid residues in the other loops or turns in the jelly-roll fold are not conserved?

RESPONSE: We agree with the reviewer. We do not have a good explanation why the D-motif is conserved and what its functional role is. Thus, we removed the speculative sentence and retained only the part where we show that this signature motif is conserved in both MPs and some of the capsid proteins.

Also, it is difficult to discuss about C-terminal and N-terminal structure, function, and evolution if the structural predictions in these parts are not accurate. As also mentioned several times in the main text, the predicted N-terminal and C-terminal structure is disordered/unstructured and I guess most likely because it failed to predict. I strongly recommend toning down results and discussion with regards to the C-terminal and the N-terminal.

RESPONSE: In the revised manuscript, we limit the discussion on the terminal regions to pointing out the differences in charge distribution between the CPs and MPs and the lengths of the extensions in MPs. Note that these considerations do not depend on the accuracy of the modeling or the actual structure of the extensions. The important adaptation of the MPs was the disappearance of the Arg-rich region (R-arm) and acquisition of the C-terminal extension, which is simply not present in the CPs.

Minor comments

1. Abstract, lines 29-31. As mentioned in a major comment, this part is too speculative.

RESPONSE: The sentence was deleted.

2. Line 227. It is not clear for me why MPs evolved from the CPs by considering their different distributions in species. MPs also seemed to find in algae viruses in this manuscript.

RESPONSE: MPs are exclusively found in a single eukaryotic kingdom, Chloroplastida, which includes plants and green algae. Note that MPs were found in viruses infecting multicellular algae, but not unicellular ones. By contrast, ssDNA and ssRNA viruses with jelly-roll capsid proteins are found in at least 9 eukaryotic kingdoms, including Chloroplastida. This is now clarified in the text.

3. Line 238. (see above). It is not obvious what should be referred.

RESPONSE: Apologies, “see above” was a relic and is eliminated in the revised version.

4. Lines 247-248. "...showed a perfect alignment...". I think it should not be perfect alignment, then two structures are the same structure.

RESPONSE: Thank you for pointing this out. The sentence has been rewritten: “Consistently, the corresponding regions of the PLRV CP and geminivirus MP structural models, including the α-helix between β-strands C and D, could be superposed (Fig. 4b).”

5. Lines 317-319. RNA-binding domains of the MPs seem not to be concluded in my reading the referred old papers. I do not think it is a good idea of combining controversy data and predicted structural data.

RESPONSE: The claims and the corresponding references were deleted.

6. Lines 322-325. This part is very speculative and no experimental data to support this idea.

RESPONSE: The sentence was removed.

7. Lines 335-341. These are all very speculative. Please tone down and make it much clear it is a speculation. Without resolving, e.g. complex structure of MPs, how to describe the impairment of the icosahedral capsid formation?

RESPONSE: We followed the reviewer's suggestion and combined the Results and Discussion. In the process, the two sentences were deleted.

8. Lines 362-386. I understand the point of the discussion regarding what likely happened to MPs after viruses adapting to plants and arthropods, however it is too lengthy and it is obscure how any of the findings in this manuscript support this hypothesis.

RESPONSE: This part has been streamlined considerably (285 down to 215 words).
