# Supplementary material for: Plant virus movement proteins originated from jelly-roll capsid proteins
Source: PLoS Biol. 2023 Jun 15;21(6):e3002157. doi: 10.1371/journal.pbio.3002157 (PMC10306228; doi:10.1371/journal.pbio.3002157)
Supplement: Attachment — Submitted filename: RESPONSES.pdf [file pbio.3002157.s017.pdf]

1. DATA POLICY:

You may be aware of the PLOS Data Policy, which requires that all data be made available without restriction: http://journals.plos.org/plosbiology/s/data-availability. For more information, please also see this editorial: http://dx.doi.org/10.1371/journal.pbio.1001797

Note that we do not require all raw data. Rather, we ask that all individual quantitative observations that underlie the data summarized in the figures and results of your paper be made available in one of the following forms:

A) Supplementary files (e.g., excel). Please ensure that all data files are uploaded as 'Supporting Information' and are invariably referred to (in the manuscript, figure legends, and the Description field when uploading your files) using the following format verbatim: S1 Data, S2 Data, etc. Multiple panels of a single or even several figures can be included as multiple sheets in one excel file that is saved using exactly the following convention: S1_Data.xlsx (using an underscore).

RESPONSE: All supplementary files were renamed as indicated.

B) Deposition in a publicly available repository. Please also provide the accession code or a reviewer link so that we may view your data before publication.

Regardless of the method selected, please ensure that you provide the individual numerical values that underlie the summary data displayed in the following figure panels as they are essential for readers to assess your analysis and to reproduce it: Figures 1AB, 3B, 5AC, and Supplementary Figures SF1, SF3, SF6.

NOTE: the numerical data provided should include all replicates AND the way in which the plotted mean and errors were derived (it should not present only the mean/average values).

Please also ensure that figure legends in your manuscript include information on where the underlying data can be found, and ensure your supplemental data file/s has a legend.

Please ensure that your Data Statement in the submission system accurately describes where your data can be found.

RESPONSE: We added the supporting information with numerical values for Figures 1AB, 3B, 5AC, and Supplementary Figures S1, S3 and S6. All supplementary files are referred to in the requested format and the location of underlying data for each figure is indicated in the corresponding figure legends.

2. Please provide a blurb which (if accepted) will be included in our weekly and monthly Electronic Table of Contents, sent out to readers of PLOS Biology, and may be used to promote your article in social media. The blurb should be about 30-40 words long and is subject to editorial changes. It should, without exaggeration, entice people to read your manuscript. It should not be redundant with the title and should not contain acronyms or abbreviations.

RESPONSE: Here is the blurb:
"Movement proteins, a signature of plant RNA and DNA viruses, enable virus cell-to-cell movement through plasmodesmata. It is shown that the most common movement proteins of the 30K superfamily

originated from jelly-roll capsid proteins of plant viruses, emphasizing the key role of exaptation in virus evolution."

3. We suggest a change in the title to make it more direct. Our suggestion: "Plant virus movement proteins originated from jelly-roll capsid proteins".

RESPONSE: Thank you for this suggestion. The title has been changed to “Plant virus movement proteins originated from jelly-roll capsid proteins”.
